# Clinical and Molecular Description of a High-Copy IncQ1 KPC-2 Plasmid Harbored by the International ST15 Klebsiella pneumoniae Clone

**DOI:** 10.1128/mSphere.00756-20

**Published:** 2020-10-07

**Authors:** Willames M. B. S. Martins, Marisa F. Nicolas, Yang Yu, Mei Li, Priscila Dantas, Kirsty Sands, Edward Portal, Luiz G. P. Almeida, Ana Tereza R. Vasconcelos, Eduardo A. Medeiros, Mark A. Toleman, Timothy R. Walsh, Ana C. Gales, Diego O. Andrey

**Affiliations:** a Department of Medical Microbiology, Division of Infection and Immunity, Cardiff University, Cardiff, United Kingdom; b Universidade Federal de São Paulo - UNIFESP, Laboratório Alerta, Division of Infectious Diseases, Department of Internal Medicine, Escola Paulista de Medicina - EPM, São Paulo, São Paulo, Brazil; c National Laboratory for Scientific Computing - LNCC, Petrópolis, Rio de Janeiro, Brazil; d National Risk Assessment Laboratory for Antimicrobial Resistance of Animal Original Bacteria, South China Agricultural University, Guangzhou, China; e Universidade Federal de São Paulo - UNIFESP, Hospital Epidemiology Committee, Hospital São Paulo, Division of Infectious Diseases, Department of Internal Medicine, Escola Paulista de Medicina - EPM, São Paulo, Brazil; f Service of Infectious Diseases, Geneva University Hospitals and Faculty of Medicine, Geneva, Switzerland; Antimicrobial Development Specialists, LLC

**Keywords:** Gram-negative bacteria, IncQ1, KPC-2, *Klebsiella pneumoniae*, ST15, bloodstream infections, carbapenemase, plasmid-mediated resistance

## Abstract

This study provides the genomic characterization and clinical description of bloodstream infections (BSI) cases due to ST15 KPC-2 producer Klebsiella pneumoniae. Six KPC-K. pneumoniae isolates were recovered in 2015 in a tertiary Brazilian hospital and were analyzed by whole-genome sequencing (WGS) (Illumina MiSeq short reads). Of these, two isolates were further analyzed by Nanopore MinION sequencing, allowing complete chromosome and plasmid circularization (hybrid assembly), using Unicycler software. The clinical analysis showed that the 30-day overall mortality for these BSI cases was high (83%). The isolates exhibited meropenem resistance (MICs, 32 to 128 mg/liter), with 3/6 isolates resistant to polymyxin B. The conjugative properties of the *bla*_KPC-2_ plasmid and its copy number were assessed by standard conjugation experiments and sequence copy number analysis. We identified in all six isolates a small (8.3-kb), high-copy-number (20 copies/cell) non-self-conjugative IncQ plasmid harboring *bla*_KPC-2_ in a non-Tn*4401* transposon. This plasmid backbone was previously reported to harbor *bla*_KPC-2_ only in Brazil, and it could be comobilized at a high frequency (10^−4^) into Escherichia coli J53 and into several high-risk K. pneumoniae clones (ST258, ST15, and ST101) by a common IncL/M helper plasmid, suggesting the potential of international spread. This study thus identified the international K. pneumoniae ST15 clone as a carrier of *bla*_KPC-2_ in a high-copy-number IncQ1 plasmid that is easily transmissible among other common *Klebsiella* strains. This finding is of concern since IncQ1 plasmids are efficient antimicrobial resistance determinant carriers across Gram-negative species. The spread of such carbapenemase-encoding IncQ1 plasmids should therefore be closely monitored.

**IMPORTANCE** In many parts of the world, carbapenem resistance is a serious public health concern. In Brazil, carbapenem resistance in *Enterobacterales* is mostly driven by the dissemination of KPC-2-producing K. pneumoniae clones. Despite being endemic in this country, only a few reports providing both clinical and genomic data are available in Brazil, which limit the understanding of the real clinical impact caused by the dissemination of different clones carrying *bla*_KPC-2_ in Brazilian hospitals. Although several of these KPC-2-producer K. pneumoniae isolates belong to the clonal complex 258 and carry Tn*4401* transposons located on large plasmids, a concomitant emergence and silent dissemination of small high-copy-number *bla*_KPC-2_ plasmids are of importance, as described in this study. Our data identify a small high-copy-number IncQ1 KPC plasmid, its clinical relevance, and the potential for conjugative transfer into several K. pneumoniae isolates, belonging to different international lineages, such as ST258, ST101, and ST15.

## Abstract (precis)

In many parts of the world, carbapenem resistance is a serious public health concern. In Brazil, carbapenem resistance in *Enterobacterales* is mostly driven by the dissemination of KPC-2-producing K. pneumoniae clones. Despite being endemic in this country, only a few reports providing both clinical and genomic data are available in Brazil, which limit the understanding of the real clinical impact caused by the dissemination of different clones carrying *bla*_KPC-2_ in Brazilian hospitals. Although several of these KPC-2-producer K. pneumoniae isolates belong to the clonal complex 258 and carry Tn*4401* transposons located on large plasmids, a concomitant emergence and silent dissemination of small high-copy-number *bla*_KPC-2_ plasmids are of importance, as described in this study. Our data identify a small high-copy-number IncQ1 KPC plasmid, its clinical relevance, and the potential for conjugative transfer into several K. pneumoniae isolates, belonging to different international lineages, such as ST258, ST101, and ST15.

## INTRODUCTION

Carbapenem resistance in *Enterobacterales* represents a serious threat to modern medicine and the global health system, as stressed by international agencies (1). KPC-producing Klebsiella pneumoniae infections are responsible for a severe burden in health care systems, particularly in North America, Latin America, Southern and Eastern Europe, Israel, and China (2). K. pneumoniae sepsis rates have been rising in recent years; according to PHE (Public Health England, including Wales and Northern Ireland), the rate of *Klebsiella* species bacteremia increased from 12 cases in 2009 to 17 cases in 2018 per 100,000 population (3).

The Brazilian Health Surveillance Agency (ANVISA) ranked K. pneumoniae as the most frequent pathogen (19.0%) causing central catheter-related bloodstream infections (CR-BSI) among adult intensive care unit (ICU) patients in 2017, with an increasing carbapenem resistance rate of 44.1% (4). This high rate is mostly due to the dissemination in Brazilian hospitals of various KPC-2-producing K. pneumoniae clones, belonging to the clonal complex (CC) 258, such as ST437 (a *tonB31* single-allele variant of ST258), ST11, and ST340. Recently, the international KPC clone ST258 (clade 2, KL107, a hybrid clone resulting from genomic recombination events between ST11 and ST442) has been identified as a main driver of KPC-2 dissemination (5–8). Other lineages include non-CC258 KPC-producing clones such as ST101, ST307, and ST16 (8). KPC-3-producing clones have been reported in Latin America, mainly in Colombia, but are not disseminated in Brazil (9).

In contrast, the K. pneumoniae ST15 clone (CC15) has rarely been associated with KPC in Latin America (10, 11). K. pneumoniae CC15 is a global clone associated with both human and animal infections, identified as an important carrier of extended-spectrum β-lactamases (ESBLs) and carbapenemases, particularly metallo-β-lactamases and OXA-48-like enzymes, worldwide (12–14). There are several reports of ST15 harboring NDM-1 in both Nepal and Pakistan (15, 16); OXA-48-like (OXA-48 and OXA-232) in China, Vietnam, Pakistan, and Spain (17–20); KPC-3 in Portugal; and KPC-2 in Bulgaria and China (21–23). The diversity of resistance determinants and plasmid backbones acquired by ST15 clones in the different study locations suggests a high capacity for horizontal acquisition of resistance. This high-risk clone has been described as a strong candidate for convergence of antimicrobial resistance (AMR) and hypervirulence, through the acquisition of hybrid plasmids, carrying both AMR and hypervirulence determinants (24).

In this study, we report the clinical and molecular characterization of a K. pneumoniae ST15 clone, associated with high mortality rates in a Brazilian hospital, including its *bla*_KPC-2_-bearing IncQ1 plasmid.

(This study was presented in part at the European Congress of Clinical Microbiology and Infectious Diseases, Amsterdam, The Netherlands, 13 to 16 April 2019, abstract O0917 [25].)

## RESULTS

### Clinical description.

Within a retrospective cohort of 165 KPC-2-producing K. pneumoniae BSI cases in a tertiary Brazilian hospital during the 2014 to 2016 period, six cases were due to isolates displaying a clonal pulsotype (data not shown) and were assigned to ST15 group by *in silico* multilocus sequence typing (MLST). The clinical description of these six cases is provided in Table 1. The patients were hospitalized in diverse wards throughout the hospital, and five out of six were admitted initially at the Emergency Department ICU. The overall 3-day and 30-day crude mortality was 20% (2/6 patients) and 85% (5/6 patients), respectively. Half of these patients presented with septic shock. There was one primary catheter-related BSI, and in the remaining cases the BSI were secondary to ventilator-acquired pneumonia (*n* = 2) or abdominal (*n* = 2) or urinary (*n* = 1) infections. Four out of six patients were treated with a triple antibiotic combination irrespective of *in vitro* susceptibility. In all six cases, the combination included polymyxin B, but the median number of *in vitro* active antimicrobials given to these patients was 1 (interquartile range [IQR], 1;2). The only surviving patient (case three), who had been admitted at the hospital with a urinary sepsis complicating an indwelling urethral catheter, was initially empirically treated with meropenem and ertapenem (dual carbapenem therapy) in association with polymyxin B.

**TABLE 1:** Clinical description of the six ST15 KPC-2-K. pneumoniae BSI cases^*a*^

Case	Bacterial isolate name	Patient age, yr (sex)^*a*^	Underlying disease	Mo/yr of infection	Source of bacteremia^*a*^	Ward(s) during hospital stay	Length of stay at bacteremia onset (days)	Septic shock	Pitt score	Empirical treatment/ targeted treatment	*In vitro* active antimicrobials (*n*)	30-day outcome^*b*^
1	P35	45 (M)	Endocarditis	May 2015	Lungs (VAP)	Cardiac surgery ICU	38	No	MD	PMB + **MEM** + AMK	2	Died (18 days)
2	P02	81 (M)	Cholangitis	May 2015	Abdominal	Emergency room ICU	19	Yes	6	**PMB** + **MEM**	0	Died (3 days)
3	P45	69 (M)	Urosepsis	September 2015	Urinary (indwelling catheter)	Emergency room	1	No	2	**PTZ**/PMB + **MEM** + **ERT**	1^*c*^	Survived
4	P16	72 (M)	Acute abdomen	September 2015	Abdominal	General ICU	23	Yes	6	**PMB** + **MEM** + AMK	1	Died (1 day)
5	P51	48 (M)	Multiple myeloma	December 2015	Lungs (VAP)	Internal medicine	16	Yes	2	**PMB** + **MEM** + AMK	1	Died (6 days)
6	P49	76 (M)	Acute myocardial infarction	December 2015	CR-BSI	Cardiac surgery	49	No	1	**CEF**/PMB + AMK	2	Died (13 days)

a Abbreviations: M, male; F, female; CR-BSI, catheter-related bloodstream infection; PMB, polymyxin B; MEM, meropenem; ERT, ertapenem; AMK, amikacin; PTZ, piperacillin-tazobactam; CEF, cefepime; VAP, associated pneumonia; MD, missing data. Bold drug abbreviations indicate *in vitro* nonsusceptibility.

b Number of days after bacteremia onset.

c In case 3, ERT and MEM were individually tested resistant, and *in vitro* double synergy was not tested.

### Antimicrobial susceptibility testing.

Antimicrobial susceptibility results revealed that all six KPC-2-producing ST15 isolates were highly resistant to meropenem (MICs, 32 to 128 mg/liter) but remained 100% susceptible to amikacin (MICs, 2 to 4 mg/liter) and ceftazidime-avibactam (MICs at 0.5 mg/liter). All isolates had tigecycline MICs of 1 mg/liter, while three isolates showed resistance to polymyxin B (MICs, 0.125 to 64 mg/liter; 50% susceptible).

### Genomic analysis of AMR and virulence determinants.

**FIG 1:**
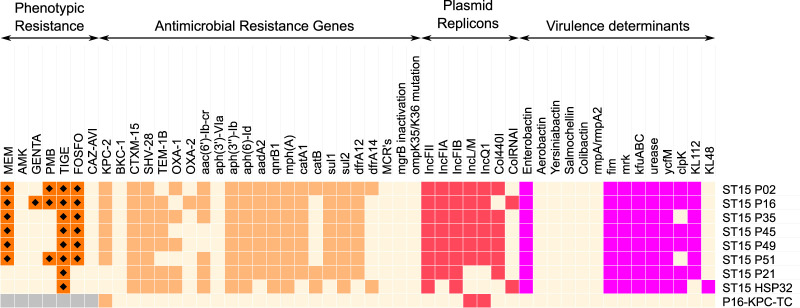
Antimicrobial resistance phenotypes and genetic profile of the six epidemic strains (P02, P16, P35, P45, P49, and P51), two ST15 comparator strains (P21 and HSP32), and a transconjugant (P16-KPC-TC). Antimicrobial susceptibility and absence of genes are indicated by light beige cells. Diamonds indicate phenotypic resistance (EUCAST breakpoints) while orange, red, and purple indicate presence of resistance, replicon, and virulence genes in the isolates, respectively. Gray indicates not determined. Abbreviations: AMK, amikacin; GENTA, gentamicin; PMB, polymyxin B; FOSFO, fosfomycin; CAZ-AVI, ceftazidime-avibactam; MEM, meropenem; TIGE, tigecycline; KL, capsular type.

The six ST15 BSI isolates (P02, P16, P35, P45, P49, and P51) and the two selected KPC-negative ST15 K. pneumoniae isolates used as comparators (P21 and HSP32) were whole-genome sequenced. Genes related to resistance, virulence determinants, and plasmid replicons are shown in Fig. 1. In the six ST15-KP isolates β-lactamases *bla*_KPC-2_, *bla*_CTX-M-15_, and *bla*_SHV-28_ were identified. The porin-encoding genes *ompK35* and *ompK36* as well as their promoter regions did not show any mutations or disruptions compared to wild-type K. pneumoniae strains, suggesting that these porins were normally expressed. The aminoglycoside resistance genes *aac(6′)-Ib-cr*, *aacA4*, *aph(3′')-Ib*, *aph(6)-Id*, and *aadA2* were also identified. No polymyxin resistance *mcr* gene or responsible mutations (*mgrB*, *phoPQ*, *pmrAB*, and *crrAB*) could be identified in the three polymyxin-resistant isolates.

The ST15 isolate genomes had type 1 (*fimA* to -*H*) and type 3 (*mrkABCDF*) fimbrial adhesion genes as well as urease (*ureA* to -*G*), outer membrane protein (*ycfM*), enterobactin siderophore (*entA* to -*F*), and *wabGHN* (lipopolysaccharide [LPS] synthesis) virulence genes. These ST15 genomes also carried the iron uptake system *kfuABC*, as previously reported for this clone. Salmochelin, yersiniabactin, aerobactin, colibactin, and *rmpA/rmpA2* hypermucoviscosity factor were not found. The six ST15 KPC-K. pneumoniae outbreak isolates harbored the KL112 (*wzi*93) capsule. The AMR and virulence determinants of the KPC-negative isolates are also displayed in Fig. 1.

### KPC-2 IncQ1 plasmid and additional plasmids.

We identified the following plasmid replicons: IncQ1, IncL/M, IncFIA, IncFII, and IncFIB (in all isolates); Col440I (in 5 isolates); and ColRNAI (in one isolate) (Fig. 1). The two KPC-negative ST15 isolates lacked IncQ1 and IncL/M replicons. The hybrid sequencing strategy (short and long reads) of isolate P35 identified 5 plasmids. By size, they were (i) pP35-IncFIB-IncFII of 248.7 kb which harbored *aadA2*, *mphA*, *catA1*, *sul1*, and *dfrA12*; (ii) pP35-IncFIA, an 85.2-kb plasmid, harboring *bla*_TEM-1B_, *bla*_CTX-M-15_, *bla*_OXA-1_, *qnrB1*, *aac(6′)-Ib-cr*, *aph(3″)-Ib*, *aph(6)-Id*, *sul2*, *catB3*, and *dfrA14*; (iii) a 53.3-kb pP35-IncL/M carrying no AMR determinant; (iv) the 8.3-kb plasmid, pP35-KPC-IncQ1, carrying *bla*_KPC-2_; and (v) a 4.1-kb pP35-Col440I lacking AMR genes.

**FIG 2:**
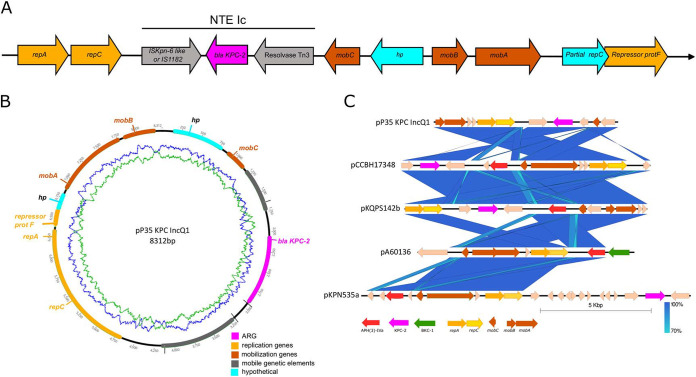
(A) Genetic context of *bla*_KPC-2_ gene. (B) Circular map of pP35-KPC-IncQ1 plasmid. (C) Alignment of IncQ1 plasmids harboring *bla*_KPC-2_ or *bla*_BKC-1_: ST15 K. pneumoniae pP35-KPC-IncQ1 (accession number CP053039), Pseudomonas aeruginosa pCCBHI17348 (accession number NOKO01000029.1), *K. quasipneumoniae* pKQPS142b (accession number CP023480), BKC-1-K. pneumoniae pA60136 (accession number KP689347), and ST340 K. pneumoniae pKPN535a (accession number MH595533).

The 8.3-kb IncQ1 plasmid harboring *bla*_KPC-2_ was identified in all isolates (depicted in Fig. 2A and B). In this plasmid, *bla*_KPC-2_ is flanked by the Tn*3* resolvase and by IS*Kpn6* (IS*1182* family) and thus belongs to NTE (non-Tn*4401*) group NTE_KPC_-Ic. pP35-KPC-IncQ1 shares a common backbone with other IncQ1 plasmids, such as pKQPS142b, identified in KPC-2-producing Klebsiella quasipneumoniae isolate KPC-142; p60136 (on BKC-1-producing K. pneumoniae A60136); and pKPN535a (on KPC-2-producing K. pneumoniae KPN535), as depicted in Fig. 2C. The IncQ1 plasmid identified in this study lacks almost all the genes necessary for self-conjugation (mating pair formation [Mpf] genes and DNA transfer and replication [Dtr] genes). The IncQ1 plasmid and *bla*_KPC-2_ were assessed at 20 copies per cell in isolate P35.

### *bla*_KPC-2_ mobilization.

To test the mobilization of *bla*_KPC-2_-IncQ1 plasmids, we performed mating-out assays first into Escherichia coli J53 and then into various K. pneumoniae recipients, belonging to high-risk clones. It showed *bla*_KPC-2_-IncQ1 conjugation at high frequency (5 × 10^−4^) into J53. Both IncQ1 and IncL/M plasmids were transferred, as verified by PCR of 10 independent transconjugants, suggesting comobilization of the IncQ plasmid. Indeed, pP35-IncL/M (and the 100% similar pP16-IncL/M) contains a complete repertoire of genes belonging to the type IV secretion system (T4SS), with both Dtr and Mpf genes (pP16-IncL/M accession number CP053039), suggesting that this 53-kb plasmid provides the Mpf machinery (T4SS) allowing comobilization of the IncQ plasmid. Subsequently, we assessed the transmissibility of the *bla*_KPC-2_-IncQ1 plasmid (using P16-KPC-TC as donor) into clinical isolates belonging to ST258, ST101, and ST15 (Table 2). Interestingly, the higher conjugation frequency (10^−6^) was observed in ST258 and ST101, in accordance with the predominant role of these clones in the global acquisition and dissemination of KPC. The expected increase in the meropenem MICs ranged from 3 to 9 log_2_ dilutions dependent upon the recipient isolate (Table 2). Altogether, these data confirm the potential for comobilization of this IncQ1 plasmid into E. coli and into several epidemiologically important K. pneumoniae clones.

**TABLE 2:** Mating-out assays using *bla*_KPC-2_ donors into several recipients^*a*^

Donor strain	Recipient name	Recipient species	Recipient ST	Recipient origin	Recipient isolation site	Transconjugant name	Frequency	MEM MIC change (R/TC)
P16	J53	E. coli	10	Lab strain		P16-KPC-TC	5 × 10^−4^	3 log^2^ dilutions (≤0.03/0.25)
P16-KPC-TC	P52	K. pneumoniae	258	Brazil	Human blood	P52-TC	1.13 × 10^−6^	4 log^2^ dilutions (2/32)
P16-KPC-TC	HSP65	K. pneumoniae	101	Brazil	Human blood	HSP65-TC	3 × 10^−6^	3 log^2^ dilutions (1/8)
P16-KPC-TC	HSP32	K. pneumoniae	15	Brazil	Human blood	HSP32-TC	8.96 × 10^−8^	5 log^2^ dilutions (≤0.03/1)
P16-KPC-TC	78623	K. pneumoniae	185	Pakistan	Human carriage	78623-TC	1.62 × 10^−7^	5 log^2^ dilutions (≤0.03/1)
P16-KPC-TC	45	K. pneumoniae	43 (SLV)	India	Human carriage	45-TC	3.5 × 10^−7^	9 log^2^ dilutions (≤0.03/16)
P16-KPC-TC	22	K. pneumoniae	858 (SLV)	India	Human carriage			
P16-KPC-TC	4W	K. pneumoniae	35	UK	Human carriage	4W-TC	3.94 × 10^−8^	4 log^2^ dilutions (≤0.03/0.5)

a Abbreviations: ST, sequence type; SLV, single locus variant; MEM, meropenem; R, recipient; TC, transconjugant.

## DISCUSSION

To date, few ST15 isolates carrying the *bla*_KPC-2_ gene have been reported (21, 22). This study reinforces our knowledge of K. pneumoniae ST15 as a multidrug-resistant clone facilitating the spread of carbapenemase genes worldwide. The clinical characteristics of the KPC-K. pneumoniae ST15-infected patients were similar to those encountered for other KPC-K. pneumoniae infections: mainly severely ill patients (high Charlson score) predominantly from ICUs. Though most isolates retained susceptibility to at least one antimicrobial prescribed for Gram-negative BSI treatment, a fatal outcome was observed in 85% of cases. The analysis of virulence factors identified the accessory iron uptake system *kfuABC*, a known invasiveness determinant generally found in ST15 lineage. Currently, there is little information available on the role of the KL112 capsule in virulence.

These ST15 isolates harbored *bla*_KPC-2_ on a small IncQ1 mobilizable high-copy-number plasmid. Interestingly *bla*_KPC-2_-bearing IncQ1 plasmids have been described only on rare occasions (8, 26–28). We show here that this plasmid carries *bla*_KPC-2_ embedded within an NTE_KPC_ element of class Ic that has successfully established itself within K. pneumoniae ST15 and spread silently in tertiary Brazilian hospitals.

**FIG 3:**
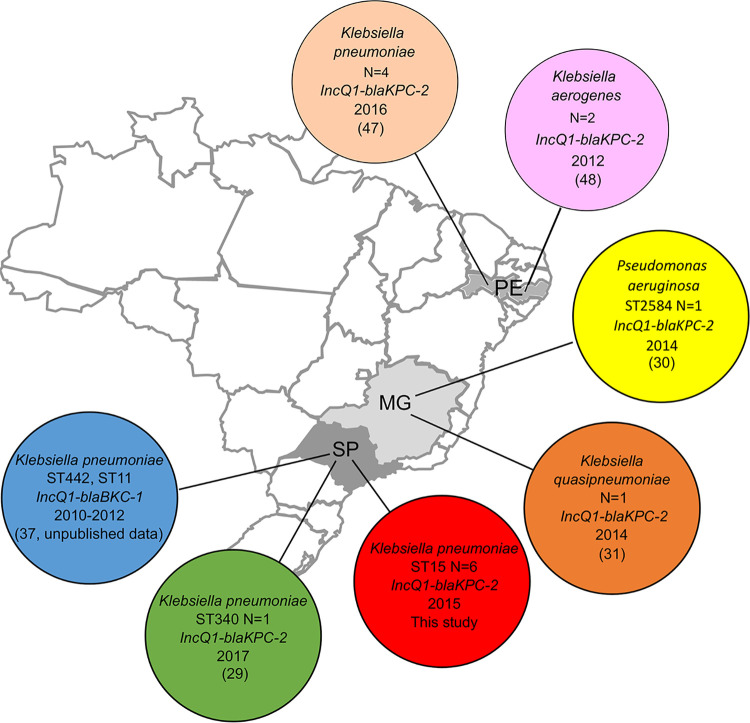
Map of reporting class A carbapenemase-produci*ng Klebsiella* species isolates harboring carbapenemase genes on IncQ1 plasmids in Brazil. SP, Sao Paulo state; MG, Minas Gerais state; PE, Pernambuco state.

Over the last 5-year period, IncQ1 plasmids carrying *bla*_KPC-2_ have been reported in several different pathogens in Brazil including Klebsiella quasipneumoniae (1 isolate, BSI), K. pneumoniae ST340 (CC258) (1 isolate, no clinical data), and Pseudomonas aeruginosa ST2584 (1 isolate, BSI), as shown in Fig. 3 (29–31). This current outbreak added a further six additional cases and suggests that IncQ1 plasmids can act as efficient *bla*_KPC-2_ carriers. The comparison of the genetic organization of IncQ1 plasmids found in geographically and temporally unrelated isolates (Fig. 2C) suggests independent parallel events rather than clonal horizontal dissemination of a unique clone-plasmid pair.

These small IncQ1 plasmids (5.1 to 14.0 kb) have been shown to have the broadest host range of any known plasmids in both Gram-negative and Gram-positive bacteria; they typically replicate independently of the host chromosome and have high copy number (32–34). This combination of high copy number, broad host range, and common comobilization means that IncQ1 plasmids are typically highly promiscuous (35). Recently, IncQ1 plasmids were reported to be involved in the *tet*(X4)-mediated tigecycline resistance dissemination in farm animals in China (36), as well as in the spread of *bla*_CMY-4_, *bla*_GES-1_, *bla*_IMP-27_, *strA*-*strB*, and *sul2* gene clusters (37–40). At the same tertiary hospital, an IncQ1 plasmid was previously described carrying the carbapenemase *bla*_BKC-1_ in K. pneumoniae isolates belonging to ST11 and ST442 (2010 to 2012) (unpublished data). We also identified a common IncL/M coresident helper plasmid that was responsible for the mobilization of these IncQ plasmids (34). Besides IncL/M plasmids, IncP, IncF, IncI, IncX, IncN, and IncW plasmids have also been described aiding IncQ1 mobilization (35).

In conclusion, we have presented here a cryptic outbreak of a K. pneumoniae ST15 clone that was carbapenem resistant due to an IncQ1 plasmid-carried *bla*_KPC-2_ gene. The outbreak resulted in several fatalities and highlights the importance of IncQ1 plasmids in the spread of the KPC carbapenemase gene. The ubiquitous presence of IncQ plasmids among both enteric and nonfermentative Gram-negative bacteria together with acquisition of KPC-2 suggests this combination of carbapenemase gene and promiscuous plasmid deserves particular attention and should be closely monitored.

## MATERIALS AND METHODS

### Study population.

The present study involves a 3-year (2014 to 2016) retrospective cohort of KPC-producing K. pneumoniae bloodstream infections (BSI), from a Brazilian public teaching hospital located in the city of São Paulo, published by our collaborative group (8). This cohort included the microbiological and genetic characterization of unique KPC-K. pneumoniae BSI adult cases. The study was approved by the Hospital São Paulo/Federal University of São Paulo (UNIFESP) Ethics Committee for Clinical Research (protocol number 1.814.158). Epidemiological and clinical data were extracted from the medical records in a standardized case form, as previously described (8).

### Isolates selection and microbiological analysis.

Six clonally related isolates, belonging to ST15, were selected for the detailed analysis presented here. In addition, two carbapenem-susceptible K. pneumoniae ST15 isolates from the same hospital collection, *bla*_KPC_ negative (HSP32 and P21), were selected for comparative genomic analysis. Isolate identification was confirmed by matrix-assisted laser desorption ionization–time of flight mass spectrometry (MALDI-TOF MS) using a Microflex LT mass spectrometer and Biotyper 3.3 software (Bruker Daltonics) according to the manufacturer’s recommendations. MICs of meropenem, amikacin, gentamicin, tigecycline, and ceftazidime-avibactam were determined by agar dilution, while the broth microdilution technique was used to determine the polymyxin B MICs. Susceptibility testing results were performed and interpreted according to European Committee on Antimicrobial Susceptibility Testing (EUCAST) recommendations (41).

### WGS and bioinformatics analysis.

The isolates were sequenced using the Illumina MiSeq platform (Illumina Inc.). DNA libraries were prepared for paired-end sequencing (2 × 300 cycles) using Nextera XT (Illumina Inc.). Quality control of raw sequence reads included FastQC (0.11.2), and adaptor trimming was performed using Trim Galore (0.4.3). K. pneumoniae genome assembly was performed using Spades (version 3.8.0), with the k-mer length increased to 127 (42). Multilocus sequence type (MLST), antimicrobial resistance (AMR) determinants, and plasmid replicons were identified using the MLST 2.0, ResFinder 3.1, and PlasmidFinder online tools (Center for Genomic Epidemiology) setting cutoff values of 90% identity and 80% minimum coverage (10 September 2018 database) (43). Virulence genes were analyzed with Geneious 10.6.1 using an in-house data set (80% minimal coverage, 75% identity) (8). Assembled genomes were submitted to the Kaptive platform, and capsular loci (KL) were determined using *Klebsiella* K locus primary as a reference (44). In addition, two isolates (P35 and P16) were selected for complete assembly (chromosome and plasmids). For these, total genomic DNA was extracted and sequenced using long-read (MinION; Oxford Nanopore Technologies), in combination with MiSeq Illumina raw short-read, hybrid *de novo* assembly using Unicycler (v0.4.0). This strategy enabled the generation of complete circularized sequences of both chromosomes and plasmids (45). Plasmid copy number was obtained based on the ratio of long reads containin*g bla*_KPC-2_ divided by the mean of chromosomal single-cop*y tonB*- and *gapA*-containing reads.

### Mating-out (conjugation) experiments.

To evaluate and compare the transferabilities of plasmid-borne *bla*_KPC-2_, conjugation assays were carried out with an ST15 donor isolate into the E. coli J53 azide-resistant strain. Subsequently, a sequence-verified J53-derived transconjugant, named P16-KPC-TC, was used as donor for a secondary conjugation set into selected K. pneumoniae isolates. Briefly, mid-log cultures of donor and recipient strains were mixed in LB broth. The mating culture was then incubated overnight at 37°C, appropriately diluted in physiological saline, and plated onto UTI agar (16636 HiCrome UTI agar; Sigma-Aldrich) containing 0.5 mg/liter meropenem for assessing the colony count. After incubation, for each conjugation, at least 5 (when available) putative transconjugant colonies were tested by restreaking onto meropenem 0.5-mg/liter UTI agar plates and the putative transconjugants were further tested by PCR for *bla*_KPC-2_. Conjugative frequency was calculated as the ratio of transconjugant CFU per donor. Isolates were considered unable to transfer *bla*_KPC-2_ into the recipient species if the transfer frequency was 10^−9^ or lower (46–48).

### Data availability.

Whole-genome sequences of the studied K. pneumoniae ST15 isolates have been deposited in the NCBI database under nucleotide accession numbers CP053035 to CP053041 and JABEPV000000000, JABEPW000000000, JABEPX000000000, JABEPY000000000, JABEPZ000000000, and JABENA000000000).

## References

[1] WHO. 2017. Guidelines for the prevention and control of carbapenem-resistant Enterobacteriaceae, Acinetobacter baumannii and Pseudomonas aeruginosa in health care facilities. World Health Organization, Geneva, Switzerland. http://www.who.int/infection-prevention/publications/guidelines-cre/en/. Accessed 18 January 2019. 29630191

[2] Munoz-Price LS, Poirel L, Bonomo RA, Schwaber MJ, Daikos GL, Cormican M, Cornaglia G, Garau J, Gniadkowski M, Hayden MK, Kumarasamy K, Livermore DM, Maya JJ, Nordmann P, Patel JB, Paterson DL, Pitout J, Villegas MV, Wang H, Woodford N, Quinn JP. 2013. Clinical epidemiology of the global expansion of Klebsiella pneumoniae carbapenemases. Lancet Infect Dis 13:785–796. doi:10.1016/S1473-3099(13)70190-7. 23969216 PMC4673667

[3] Public Health England. 2020. Klebsiella spp. bacteraemia: voluntary surveillance. Health Protection Report 14(1). https://www.gov.uk/government/publications/klebsiella-spp-bacteraemia-voluntary-surveillance.

[4] ANVISA Brazilian Agencia Nacional de Vigilancia Sanitaria. 2017. Boletim de segurança do paciente e qualidade em serviços de saúde no. 17: avaliação dos indicadores nacionals das infecções relacionadas à assistência à saúde (IRAS) e resistência microbiana do ano de 2017. https://app.powerbi.com/view?r=eyJrIjoiZTFiOGRhOTYtYzZjOS00NmZmLWE5MWUtN2RkNDhiZGJiOGE1IiwidCI6ImI2N2FmMjNmLWMzZjMtNGQzNS04MGM3LWI3MDg1ZjVlZGQ4MSJ9. Accessed 10 April 2020.

[5] Andrade LN, Curiao T, Ferreira JC, Longo JM, Climaco EC, Martinez R, Bellissimo-Rodrigues F, Basile-Filho A, Evaristo MA, Del Peloso PF, Ribeiro VB, Barth AL, Paula MC, Baquero F, Canton R, Darini AL, Coque TM. 2011. Dissemination of *bla*_KPC-2_ by the spread of *Klebsiella pneumoniae* clonal complex 258 clones (ST258, ST11, ST437) and plasmids (IncFII, IncN, IncL/M) among *Enterobacteriaceae* species in Brazil. Antimicrob Agents Chemother 55:3579–3583. doi:10.1128/AAC.01783-10. 21576442 PMC3122403

[6] Pereira PS, de Araujo CF, Seki LM, Zahner V, Carvalho-Assef AP, Asensi MD. 2013. Update of the molecular epidemiology of KPC-2-producing Klebsiella pneumoniae in Brazil: spread of clonal complex 11 (ST11, ST437 and ST340). J Antimicrob Chemother 68:312–316. doi:10.1093/jac/dks396. 23070735

[7] Chen L, Mathema B, Pitout JD, DeLeo FR, Kreiswirth BN. 2014. Epidemic *Klebsiella pneumoniae* ST258 is a hybrid strain. mBio 5:e01355-14. doi:10.1128/mBio.01355-14. 24961694 PMC4073492

[8] Andrey DO, Dantas P, Martins WBS, Marques de Carvalho F, Gonzaga LA, Sands K, Portal E, Sauser J, Cayo R, Nicolas MF, Vasconcelos ATR, Medeiros EA, Walsh TR, Gales AC. 2019. An emerging clone, KPC-2-producing Klebsiella pneumoniae ST16, associated with high mortality rates in a CC258 endemic setting. Clin Infect Dis doi:10.1093/cid/ciz1095. PMC7583420 31712802

[9] Rojas LJ, Weinstock GM, De La Cadena E, Diaz L, Rios R, Hanson BM, Brown JS, Vats P, Phillips DS, Nguyen H, Hujer KM, Correa A, Adams MD, Perez F, Sodergren E, Narechania A, Planet PJ, Villegas MV, Bonomo RA, Arias CA. 2017. An analysis of the epidemic of Klebsiella pneumoniae carbapenemase-producing K. pneumoniae: convergence of two evolutionary mechanisms creates the “perfect storm.” J Infect Dis 217:82–92. doi:10.1093/infdis/jix524. 29029188 PMC5853647

[10] Bialek-Davenet S, Criscuolo A, Ailloud F, Passet V, Jones L, Delannoy-Vieillard AS, Garin B, Le Hello S, Arlet G, Nicolas-Chanoine MH, Decre D, Brisse S. 2014. Genomic definition of hypervirulent and multidrug-resistant Klebsiella pneumoniae clonal groups. Emerg Infect Dis 20:1812–1820. doi:10.3201/eid2011.140206. 25341126 PMC4214299

[11] Cejas D, Elena A, Nunez DG, Platero PS, De Paulis A, Magarinos F, Alfonso C, Berger MA, Canigia LF, Gutkind G, Radice M. 2019. Changing epidemiology of KPC producing Klebsiella pneumoniae in Argentina: emergence of hypermucoviscous ST25 and high risk clone ST307. J Glob Antimicrob Resist 18:238–242. doi:10.1016/j.jgar.2019.06.005. 31202977

[12] Wyres KL, Lam MMC, Holt KE. 2020. Population genomics of Klebsiella pneumoniae. Nat Rev Microbiol 18:344–359. doi:10.1038/s41579-019-0315-1. 32055025

[13] Zhou K, Lokate M, Deurenberg RH, Tepper M, Arends JP, Raangs EG, Lo-Ten-Foe J, Grundmann H, Rossen JW, Friedrich AW. 2016. Use of whole-genome sequencing to trace, control and characterize the regional expansion of extended-spectrum beta-lactamase producing ST15 Klebsiella pneumoniae. Sci Rep 6:20840. doi:10.1038/srep20840. 26864946 PMC4749987

[14] Rodrigues C, Machado E, Ramos H, Peixe L, Novais A. 2014. Expansion of ESBL-producing Klebsiella pneumoniae in hospitalized patients: a successful story of international clones (ST15, ST147, ST336) and epidemic plasmids (IncR, IncFIIK). Int J Med Microbiol 304:1100–1108. doi:10.1016/j.ijmm.2014.08.003. 25190354

[15] Chung The H, Karkey A, Pham Thanh D, Boinett CJ, Cain AK, Ellington M, Baker KS, Dongol S, Thompson C, Harris SR, Jombart T, Le Thi Phuong T, Tran Do Hoang N, Ha Thanh T, Shretha S, Joshi S, Basnyat B, Thwaites G, Thomson NR, Rabaa MA, Baker S. 2015. A high-resolution genomic analysis of multidrug-resistant hospital outbreaks of Klebsiella pneumoniae. EMBO Mol Med 7:227–239. doi:10.15252/emmm.201404767. 25712531 PMC4364942

[16] Ferreira A, Carvalho M, Sands K, Thomson K, Portal E, Mathias J, Nieto M, Hender T, Dyer C, Milton R, Iregbu K, Mazarati JB, Chan G, Mehtar S, Zahra R, Basu S, Saha S, Jones L, Walsh TR. 2019. Burden of antibiotic resistance in neonates from developing societies (BARNARDS): Klebsiella pneumoniae in neonatal sepsis, abstr O0873. 29th ECCMID, Amsterdam, Netherlands, 13 to 16 April 2019.

[17] Madueno A, Gonzalez Garcia J, Fernandez-Romero S, Oteo J, Lecuona M. 2017. Dissemination and clinical implications of multidrug-resistant Klebsiella pneumoniae isolates producing OXA-48 in a Spanish hospital. J Hosp Infect 96:116–122. doi:10.1016/j.jhin.2017.02.024. 28395861

[18] Shu L, Dong N, Lu J, Zheng Z, Hu J, Zeng W, Sun Q, Chan EW, Zhou H, Hu F, Chen S, Zhang R. 2018. Emergence of OXA-232 carbapenemase-producing Klebsiella pneumoniae that carries a pLVPK-like virulence plasmid among elderly patients in China. Antimicrob Agents Chemother 63:e02246-18. doi:10.1128/AAC.02246-18. PMC6395905 30559135

[19] Yin D, Dong D, Li K, Zhang L, Liang J, Yang Y, Wu N, Bao Y, Wang C, Hu F. 2017. Clonal dissemination of OXA-232 carbapenemase-producing Klebsiella pneumoniae in neonates. Antimicrob Agents Chemother 61:e00385-17. doi:10.1128/AAC.00385-17. 28533245 PMC5527636

[20] Heinz E, Ejaz H, Bartholdson Scott J, Wang N, Gujaran S, Pickard D, Wilksch J, Cao H, Haq IU, Dougan G, Strugnell RA. 2019. Resistance mechanisms and population structure of highly drug resistant Klebsiella in Pakistan during the introduction of the carbapenemase NDM-1. Sci Rep 9:2392. doi:10.1038/s41598-019-38943-7. 30787414 PMC6382945

[21] Vubil D, Figueiredo R, Reis T, Canha C, Boaventura L, DA Silva GJ. 2017. Outbreak of KPC-3-producing ST15 and ST348 Klebsiella pneumoniae in a Portuguese hospital. Epidemiol Infect 145:595–599. doi:10.1017/S0950268816002442. 27788691 PMC9507641

[22] Markovska R, Stoeva T, Schneider I, Boyanova L, Popova V, Dacheva D, Kaneva R, Bauernfeind A, Mitev V, Mitov I. 2015. Clonal dissemination of multilocus sequence type ST15 KPC-2-producing Klebsiella pneumoniae in Bulgaria. APMIS 123:887–894. doi:10.1111/apm.12433. 26303718

[23] Qi Y, Wei Z, Ji S, Du X, Shen P, Yu Y. 2011. ST11, the dominant clone of KPC-producing Klebsiella pneumoniae in China. J Antimicrob Chemother 66:307–312. doi:10.1093/jac/dkq431. 21131324

[24] Lam MMC, Wyres KL, Wick RR, Judd LM, Fostervold A, Holt KE, Lohr IH. 2019. Convergence of virulence and MDR in a single plasmid vector in MDR Klebsiella pneumoniae ST15. J Antimicrob Chemother 74:1218–1222. doi:10.1093/jac/dkz028. 30770708 PMC6477991

[25] Martins WB, Yu Y, Dantas P, Sands K, Portal E, Medeiros EA, Toleman MA, Walsh TR, Gales A, Andrey D. 2019. Fatal bloodstream infections due to a ST15 *Klebsiella pneumoniae* carrying *bla*KPC-2 in a (non-Tn*4401*) mobilisable IncQ1 high copy plasmid, abstr. O0917. 29th ECCMID, Amsterdam, Netherlands, 13 to 16 April 2019.

[26] Ramos PI, Picao RC, Almeida LG, Lima NC, Girardello R, Vivan AC, Xavier DE, Barcellos FG, Pelisson M, Vespero EC, Medigue C, Vasconcelos AT, Gales AC, Nicolas MF. 2014. Comparative analysis of the complete genome of KPC-2-producing Klebsiella pneumoniae Kp13 reveals remarkable genome plasticity and a wide repertoire of virulence and resistance mechanisms. BMC Genomics 15:54. doi:10.1186/1471-2164-15-54. 24450656 PMC3904158

[27] Cerdeira LT, Cunha MPV, Francisco GR, Bueno MFC, Araujo BF, Ribas RM, Gontijo-Filho PP, Knobl T, de Oliveira Garcia D, Lincopan N. 2017. IncX3 plasmid harboring a non-Tn4401 genetic element (NTEKPC) in a hospital-associated clone of KPC-2-producing Klebsiella pneumoniae ST340/CG258. Diagn Microbiol Infect Dis 89:164–167. doi:10.1016/j.diagmicrobio.2017.06.022. 28807400

[28] Campos PA, Fuga B, Cerdeira LT, Ferreira ML, Dias VL, Machado LG, Rossi I, Lincopan N, Gontijo-Filho PP, Ribas RM. 2019. Early dissemination of IncQ1 plasmids in KPC-2-producing Klebsiella pneumoniae CG258. Microb Drug Resist 25:1257–1259. doi:10.1089/mdr.2019.0123. 31188044

[29] Cerdeira LT, Lam MMC, Wyres KL, Wick RR, Judd LM, Lopes R, Ribas RM, Morais MM, Holt KE, Lincopan N. 2019. Small IncQ1 and Col-like plasmids harboring bla KPC-2 and non-Tn4401 elements (NTEKPC-IId) in high-risk lineages of Klebsiella pneumoniae CG258. Antimicrob Agents Chemother 63:e02140-18. doi:10.1128/AAC.02140-18. 30602517 PMC6395902

[30] de Oliveira Santos IC, Albano RM, Asensi MD, D’Alincourt Carvalho-Assef AP. 2018. Draft genome sequence of KPC-2-producing Pseudomonas aeruginosa recovered from a bloodstream infection sample in Brazil. J Glob Antimicrob Resist 15:99–100. doi:10.1016/j.jgar.2018.08.021. 30172833

[31] Nicolas MF, Ramos PIP, Marques de Carvalho F, Camargo DRA, de Fatima Morais Alves C, Loss de Morais G, Almeida LGP, Souza RC, Ciapina LP, Vicente ACP, Coimbra RS, Ribeiro de Vasconcelos AT. 2018. Comparative genomic analysis of a clinical isolate of Klebsiella quasipneumoniae subsp. similipneumoniae, a KPC-2 and OKP-B-6 beta-lactamases producer harboring two drug-resistance plasmids from southeast Brazil. Front Microbiol 9:220. doi:10.3389/fmicb.2018.00220. 29503635 PMC5820359

[32] Meyer R. 2009. Replication and conjugative mobilization of broad host-range IncQ plasmids. Plasmid 62:57–70. doi:10.1016/j.plasmid.2009.05.001. 19465049 PMC2752045

[33] Francia MV, Varsaki A, Garcillan-Barcia MP, Latorre A, Drainas C, de la Cruz F. 2004. A classification scheme for mobilization regions of bacterial plasmids. FEMS Microbiol Rev 28:79–100. doi:10.1016/j.femsre.2003.09.001. 14975531

[34] Rawlings DE, Tietze E. 2001. Comparative biology of IncQ and IncQ-like plasmids. Microbiol Mol Biol Rev 65:481–496. doi:10.1128/MMBR.65.4.481-496.2001. 11729261 PMC99038

[35] Loftie-Eaton W, Rawlings DE. 2012. Diversity, biology and evolution of IncQ-family plasmids. Plasmid 67:15–34. doi:10.1016/j.plasmid.2011.10.001. 22037393

[36] Sun J, Chen C, Cui CY, Zhang Y, Liu X, Cui ZH, Ma XY, Feng Y, Fang LX, Lian XL, Zhang RM, Tang YZ, Zhang KX, Liu HM, Zhuang ZH, Zhou SD, Lv JN, Du H, Huang B, Yu FY, Mathema B, Kreiswirth BN, Liao XP, Chen L, Liu YH. 2019. Plasmid-encoded tet(X) genes that confer high-level tigecycline resistance in Escherichia coli. Nat Microbiol 4:1457–1464. doi:10.1038/s41564-019-0496-4. 31235960 PMC6707864

[37] Nicoletti AG, Marcondes MF, Martins WM, Almeida LG, Nicolas MF, Vasconcelos AT, Oliveira V, Gales AC. 2015. Characterization of BKC-1 class A carbapenemase from Klebsiella pneumoniae clinical isolates in Brazil. Antimicrob Agents Chemother 59:5159–5164. doi:10.1128/AAC.00158-15. 26055384 PMC4538461

[38] Kotsakis SD, Tzouvelekis LS, Lebessi E, Doudoulakakis A, Bouli T, Tzelepi E, Miriagou V. 2015. Characterization of a mobilizable IncQ plasmid encoding cephalosporinase CMY-4 in Escherichia coli. Antimicrob Agents Chemother 59:2964–2966. doi:10.1128/AAC.05017-14. 25691650 PMC4394814

[39] Poirel L, Carattoli A, Bernabeu S, Bruderer T, Frei R, Nordmann P. 2010. A novel IncQ plasmid type harbouring a class 3 integron from Escherichia coli. J Antimicrob Chemother 65:1594–1598. doi:10.1093/jac/dkq166. 20525990

[40] Yau S, Liu X, Djordjevic SP, Hall RM. 2010. RSF1010-like plasmids in Australian Salmonella enterica serovar Typhimurium and origin of their sul2-strA-strB antibiotic resistance gene cluster. Microb Drug Resist 16:249–252. doi:10.1089/mdr.2010.0033. 20617928

[41] European Committee on Antimicrobial Susceptibility Testing. 2020. Clinical breakpoints and dosing of antibiotics. http://www.eucast.org/clinical_breakpoints/. Accessed 20 June 2020.

[42] Nurk S, Bankevich A, Antipov D, Gurevich AA, Korobeynikov A, Lapidus A, Prjibelski AD, Pyshkin A, Sirotkin A, Sirotkin Y, Stepanauskas R, Clingenpeel SR, Woyke T, McLean JS, Lasken R, Tesler G, Alekseyev MA, Pevzner PA. 2013. Assembling single-cell genomes and mini-metagenomes from chimeric MDA products. J Comput Biol 20:714–737. doi:10.1089/cmb.2013.0084. 24093227 PMC3791033

[43] Zankari E, Hasman H, Cosentino S, Vestergaard M, Rasmussen S, Lund O, Aarestrup FM, Larsen MV. 2012. Identification of acquired antimicrobial resistance genes. J Antimicrob Chemother 67:2640–2644. doi:10.1093/jac/dks261. 22782487 PMC3468078

[44] Wyres KL, Wick RR, Gorrie C, Jenney A, Follador R, Thomson NR, Holt KE. 2016. Identification of Klebsiella capsule synthesis loci from whole genome data. Microb Genom 2:e000102. doi:10.1099/mgen.0.000102. 28348840 PMC5359410

[45] Wick RR, Judd LM, Gorrie CL, Holt KE. 2017. Unicycler: resolving bacterial genome assemblies from short and long sequencing reads. PLoS Comput Biol 13:e1005595. doi:10.1371/journal.pcbi.1005595. 28594827 PMC5481147

[46] Yu Y, DO A, Yang RS, Sands K, Tansawai U, Li M, Portal E, Gales AC, Niumsup PR, Sun J, Liao X, Liu YH, Walsh TR. 2020. A Klebsiella pneumoniae strain co-harbouring mcr-1 and mcr-3 from a human in Thailand. J Antimicrob Chemother 75:2372–2374. doi:10.1093/jac/dkaa133. 32294160

[47] Lima GJ, Scavuzzi AML, Beltrao EMB, Firmo EF, Oliveira EM, Oliveira SR, Rezende AM, Lopes ACS. 2020. Identification of plasmid IncQ1 and NTE_KPC_-IId harboring *bla*_KPC-2_ in isolates from *Klebsiella pneumoniae* infections in patients from Recife-PE, Brazil. Rev Soc Bras Med Trop 53:e20190526. doi:10.1590/0037-8682-0526-2019. 32578705 PMC7310361

[48] Bispo Beltrao EM, de Oliveira EM, Dos Santos Vasconcelos CR, Cabral AB, Rezende AM, Souza Lopes AC. 2020. Multidrug-resistant *Klebsiella aerogenes* clinical isolates from Brazil carrying IncQ1 plasmids containing the *bla*_KPC-2_ gene associated with non-Tn*4401* elements (NTE_KPC_-IId). J Glob Antimicrob Resist 22:43–44. doi:10.1016/j.jgar.2020.05.001. 32445743

